# Leiomyosarcoma of the Right Gonadal Vein: Review of the Approach and Prognostic in a Rare Case

**DOI:** 10.1155/2019/4042689

**Published:** 2019-01-22

**Authors:** Noa de la Fuente, Manuel Rodríguez Blanco, Gemma Cerdán, Antonio Moral, Vicenç Artigas

**Affiliations:** Department of General Surgery, Hospital de la Santa Creu I Sant Pau, San Quintín, 89, 08041 Barcelona, Spain

## Abstract

**Background:**

Venous leiomyosarcoma is a mesenchymal tumour that represents 5-7% of soft tissue sarcomas. It originates in the smooth muscle cells of the vessel wall and is frequently located in the inferior vena cava. Primary leiomyosarcomas of the gonadal vein are rare, with only 10 cases reported in the literature.

**Case report:**

We present the case of a 51-year-old woman diagnosed with a right retroperitoneal mass by computed tomography (CT). The differential diagnosis was between a neurogenic tumour and a mesodermic tumour. The tumour was dissected from the vena cava and right ureter by laparoscopy without performing resection en bloc. Histologic examination of the surgical specimen showed a high-grade leiomyosarcoma of the right gonadal vein. The postoperative course was uneventful. Three cycles of chemotherapy with epirubicin-ifosfamide were performed.

**Discussion and conclusions:**

Venous leiomyosarcoma is an aggressive tumour, and prognosis is poor due to haematogenous spread. No chemotherapy or radiotherapy has yet proven effective in improving survival, and complete surgical excision is currently considered to offer the best chance of cure. Despite the more conservative treatment approach used in the present case, the patient is alive three years after surgery and has a good quality of life. Although it was not used in this patient, the standard procedure for optimal survival is resection en bloc.

## 1. Introduction

Venous leiomyosarcoma is a rare mesenchymal tumour that accounts for 5-7% of soft tissue sarcomas [1]. Arising from smooth muscle cells of the vessel wall, it is most commonly located in the inferior cava (50% of cases), but it may also occur in renal, mesenteric, hepatic, saphenous, or gonadal veins. These leiomyosarcomas are more common in women than in men, and they are generally found in the older population (mainly postmenopausal women) [1, 2]. Approximately 200 cases of leiomyosarcoma in the inferior cava vein have been reported to date [1–6].

Histologic examination is similar to that for leiomyoma and usually shows sweeping bundles of spindle-shaped cells, eosinophil cytoplasm, perinuclear vacuoles, and different mitotic figures. Areas of necrosis and mononuclear giant cells are observed. Immunohistochemistry is positive for desmin, vimentin, and smooth cell actin, but not for S-100 protein or neuron-specific enolase [1, 2, 4, 7].

Here, we report a case of a leiomyosarcoma of the right gonadal vein. We describe the surgical procedure, the pathologic findings, and the clinical course, and we review the literature about this rare disease.

## 2. Case Report

A 51-year-old woman was presented with complaints of diarrhoea for 3 years. She had no medical history. Colonoscopy revealed slight extrinsic compression of the hepatic angle. CT examination showed a right retroperitoneal mass of 65 × 60 × 90 mm. The well-defined solid lesion was located in the right retroperitoneum, posterior and inferior to the duodenum, on the right side of the cava, and anterior to and on the left side of the right kidney. Based on these findings, the differential diagnosis was between a neurogenic tumour and a mesodermal tumour.

Blood tests for tumoural markers, chromogranin A and urine metanephrines, were negative.

At a sarcoma multidisciplinary meeting, surgery was favoured over biopsy. Due to the inconclusive diagnosis, a conservative laparoscopic approach treatment was decided.

With the patient placed in left lateral decubitus position, four trocars were placed in a semicircular line in the right hemiabdomen. The right angle of the colon was mobilized to locate the tumour in the right side of the duodenum and the cava. The tumour was then dissected by ultrasonic shears and by blunt dissection from the cava; the right gonadal vein and the ureter were found to be in contact with the tumour but without infiltration.

Dissection indicated the tumour arose from the gonadal vein, and this was therefore clipped and divided. The tumour was maintained and completely removed by an accessory incision in the right flank.

En bloc resection was ruled out in view of the uncertain diagnosis. Postsurgical recovery was uneventful. Oral intake was started on the fourth day because she had nausea and vomiting in the immediate postoperative period. She returns slowly to a regular diet and was discharged on the ninth postoperative day.

The histological report confirmed a high-grade leiomyosarcoma (grade 2), with areas of focal necrosis, dystrophic calcification, and positive resection margins (R1). The tumour was described as a fusocelular sarcoma with crossed bundles and high pleomorphism. The mitotic index was less than 9 on 10 high-power fields. In some sections, the morphology seemed to arise from a vessel wall. Immunohistochemistry was positive for alpha actin, desmin, and caldesmon and negative for CD117 and S-100 protein.

The case was again evaluated at a sarcoma multidisciplinary meeting. After the diagnosis was confirmed, it was decided to administer three cycles of adjuvant chemotherapy (epirubicin-ifosfamide) and 50 Gy radiotherapy. The only medical complication was the raised levels of hepatic transaminases. The follow-up was done by blood tests every 6 months and computed tomography (CT) with endovenous contrast every year.

Three years after surgery, although the recommended treatment with en bloc excision with wide nonaffected margins was not performed, the patient is still alive, free of disease, and has a good quality of life. She has not had a recurrence of the disease.

## 3. Discussion

A venous leiomyosarcoma should be suspected in multiple leiomyomas and in cases of a rapidly growing pelvic or fibroid mass [2–5, 7]. The tumour may be associated with abdominal pain, but patients are mainly asymptomatic, particularly if the lesion originates in the retroperitoneal space, in which case diagnosis is delayed and the prognosis is worse [1, 2]. Diagnosis is incidental in almost one-third of cases. Differential diagnosis should be done with other type of tumours like retroperitoneal tumours and renal carcinoma with vein infiltration.

The gold standard imaging test for presurgical diagnosis is computed tomography (CT) with endovenous contrast to assess the extent of the disease and guide the management strategy. CT outlines the tumour boundaries before performing a surgical procedure [1–6]. CT reveals large masses with heterogeneous contrast, cystic and necrotic components, and vascular hypertrophy [3, 5]. An MRI may also be useful for the diagnosis if a venous leiomyosarcoma is suspected [2].

Surgery is the first option because survival rates are higher than those reported for other approaches. Radical surgical criteria should be considered. En bloc resection with negative margins has been reported to achieve the best prognosis, with a 5-year survival rate in 33-68% of the cases [1, 2, 8].

Over fifty percent of patients with complete macroscopic resection have shown recurrent disease despite early surgical intervention and die due to disease progression. Because of the low incidence of this tumour, there is a lack not only of standard protocols of chemotherapy and radiotherapy but also of clinical trials showing any increase in survival. Some authors recommend radiotherapy based on the high risk of local recurrence, but its usefulness should be determined in prospective randomized trials [1, 2, 4–8]. Moreover, systemic chemotherapy should be considered for large high-grade tumours, recurrent tumours, and metastatic disease. Follow-up for local recurrence and metastasis should be performed regularly over the first 5 years.

In a review of the literature concerning venous leiomyosarcoma management, we found 30 reviews on vena cava leiomyosarcomas from the last 40 years but only one review, by Gage et al. in 2012, on noncava leiomyosarcomas. In their study of 143 cases, the authors reported that this tumour was most frequently diagnosed in women aged 60-69 and that the most common tumour site was the renal vein. They found metastases in 12% of the cases at diagnosis. With regard to treatment, 95% of the patients were treated surgically, and radical surgery involving adjacent organs was most commonly performed. Fifty percent of patients received adjuvant therapy (chemotherapy or radiotherapy). In terms of survival, 32% of patients were alive four years later.

In the present case, we decided to perform conservative surgery because of the doubtful diagnosis. We used a laparoscopic approach and preserved some adjacent structures. It is of particular note in this case that despite the positive margins at laparoscopy, the patient is alive and free of disease three years after the procedure.

It should be kept in mind that whenever possible and if the diagnosis is clearly a sarcoma, it is mandatory to perform en bloc radical surgery in order to obtain free margins and better survival. The procedure should not be performed using a laparoscopic approach if free margins are not feasible. We do recommend an open approach and en bloc resection if the tumour is really indicative of leiomyosarcoma. When the diagnosis of this sarcoma is doubtful or if the tumour is unresectable, a biopsy should be performed prior to surgery to determine the optimal management [9].

## 4. Conclusion

Leiomyosarcoma of the gonadal vein is a rare mesenchymal tumour that is normally diagnosed as a retroperitoneal mass. Symptoms are usually nonspecific so diagnosis is delayed. The recommended treatment is en bloc resection. Although margins may be negative at surgery, the prognosis is poor due to the possibility of high haematogenous spread and local recurrence. In case of clinical suspicion of this sarcoma, imaging techniques are essential to aid diagnosis and to prioritize surgery in resectable cases so as to optimize survival.
